# CRY Drives Cyclic CK2-Mediated BMAL1 Phosphorylation to Control the Mammalian Circadian Clock

**DOI:** 10.1371/journal.pbio.1002293

**Published:** 2015-11-12

**Authors:** Teruya Tamaru, Mitsuru Hattori, Kousuke Honda, Yasukazu Nakahata, Paolo Sassone-Corsi, Gijsbertus T. J. van der Horst, Takeaki Ozawa, Ken Takamatsu

**Affiliations:** 1 Department of Physiology and Advanced Research Center for Medical Science, Toho University School of Medicine, Tokyo, Japan; 2 Department of Chemistry, The University of Tokyo, Tokyo, Japan; 3 Laboratory of Gene Regulation Research, Graduate School of Biological Sciences, Nara Institute of Science and Technology, Ikoma, Japan; 4 Department of Biological Chemistry, University of California, Irvine, Irvine, California, United States of America; 5 Department of Genetics, Erasmus University Medical Center, Rotterdam, The Netherlands; Washington University Medical School, UNITED STATES

## Abstract

Intracellular circadian clocks, composed of clock genes that act in transcription-translation feedback loops, drive global rhythmic expression of the mammalian transcriptome and allow an organism to anticipate to the momentum of the day. Using a novel clock-perturbing peptide, we established a pivotal role for casein kinase (CK)-2-mediated circadian BMAL1-Ser90 phosphorylation (BMAL1-P) in regulating central and peripheral core clocks. Subsequent analysis of the underlying mechanism showed a novel role of CRY as a repressor for protein kinase. Co-immunoprecipitation experiments and real-time monitoring of protein–protein interactions revealed that CRY-mediated periodic binding of CK2β to BMAL1 inhibits BMAL1-Ser90 phosphorylation by CK2α. The FAD binding domain of CRY1, two C-terminal BMAL1 domains, and particularly BMAL1-Lys537 acetylation/deacetylation by CLOCK/SIRT1, were shown to be critical for CRY-mediated BMAL1–CK2β binding. Reciprocally, BMAL1-Ser90 phosphorylation is prerequisite for BMAL1-Lys537 acetylation. We propose a dual negative-feedback model in which a CRY-dependent CK2-driven posttranslational BMAL1–P-BMAL1 loop is an integral part of the core clock oscillator.

## Abstract 2

Cryptochrome-dependent cyclic phosphorylation of BMAL1 by CK2 serves as an integral gear in the mammalian clockwork that drives daily rhythms in physiology and behavior. Read the Synopsis.

## Author Summary

The circadian system imposes daily rhythms on behavior, physiology, and metabolism and allows organisms to anticipate daily recurring changes in the environment. Circadian clocks are composed of clock genes, acting in transcription–translation feedback loops. At the heart of this molecular oscillator are the BMAL1–CLOCK transcription factors that drive expression of *Cryptochrome* (*Cry*) and *Period* genes, which in turn encode inhibitors of BMAL1–CLOCK–driven transcription. Phosphorylation and other posttranslational modifications also specify the activity and stability of clock proteins. Here, we unveiled the key mechanism underlying the rhythmic phosphorylation of BMAL1 at Ser90 by Casein Kinase-2 alpha (CK2α). Performing live monitoring of protein–protein interactions, we show that CRY proteins facilitate cyclic BMAL1–CK2β binding and subsequent cyclic inactivation of CK2α-mediated BMAL1-S90 phosphorylation; lack of this cyclic event abolishes circadian rhythmicity. We propose a dual negative-feedback model in which CRY proteins not only act as inhibitors of BMAL1–CLOCK transcription but also of BMAL1-S90 phosphorylation.

## Introduction

The mammalian circadian system orchestrates a wide variety of metabolic, physiological, and behavioral rhythms through intracellular clockworks, present in the neurons of the suprachiasmatic nuclei (SCN) and in virtually all other cells and tissues [1]. At the heart of circadian time keeping is a molecular core oscillator consisting of a set of transcription factors (clock proteins) that operate in transcriptional–translational feedback loops [1–5] and drive rhythmic expression of approximately 10% of the mammalian transcriptome [6]. The BMAL1–CLOCK heterodimer is the primary genome-wide driver for transcription of clock genes (including three *period* [*Per1*, *2*, and *3*] and two *cryptochrome* [*Cry1* and *2*] clock genes) and clock-controlled output genes (CCGs) via binding to E-box containing promoters [7,8]. Periodic association of Cryptochrome (CRY)–PER complexes with BMAL1–CLOCK after the nuclear translocation of CRY–PER is facilitated by CLOCK-mediated BMAL1 acetylation, which represses the expression of E-box clock (controlled) genes and accounts for the negative limb of the circadian feedback loop [4]. The BMAL1–CLOCK complex also drives transcription of the retinoic acid receptor related orphan receptor α (*Rorα*) and nuclear receptor subfamily 1, group D, member 1 (*NR1D1* or *Rev-erbα*) orphan nuclear receptor genes. RORα and REV-ERBα, after binding to RORE elements in the *Bmal1* promoter, activate and repress *Bmal1* transcription and stabilize the circadian oscillator by driving cyclic expression of *Bmal1* [9]. Importantly, to date, only BMAL1- and CRY1/2-deficient mice exhibit immediate and complete loss in circadian rhythms [7,10].

Posttranslational modification represents an essential control feature of molecular oscillators in both prokaryotic and eukaryotic organisms [11] can specify longevity, activity, stability, and subcellular localization of core clock proteins. Indeed, mammalian clock proteins are important targets of posttranslational modification events [3–5,12], and their rhythmic phosphorylation appears to be a critical step for clock function [8,13–15]. For instance, *Rev-erbα* knockout mice (in which cyclic *Bmal1* transcription is blunted, but rhythmic BMAL1 modification probably remains intact) still exhibit circadian rhythms [16], suggesting that cyclic BMAL1 modification is more critical in clock machinery than cyclic transcription.

Casein kinase (CK)-2α [17] rhythmically phosphorylates BMAL1 and is pivotal for regulating the mammalian circadian clock [12]. Depletion of CK2α and/or mutation of the CK2-phosphorylation site in BMAL1 (Ser90), results in impaired circadian nuclear BMAL1 accumulation and impairment of BMAL1–CLOCK–driven rhythmic CCG expression [12]. CK2 is shown to be an indispensable component of the mammalian molecular clock [12,18,19]. CK2 also phosphorylates PER2 to control PER2 degradation, and depletion of CK2 results in impaired clock gene oscillation because of lower amplitude of the expression rhythm and an extended period.

To date, the molecular mechanism underlying rhythmic mammalian clock protein phosphorylation remains elusive. Previously, we have reported circadian BMAL1 phosphorylation at Ser90 by CK2 [12], which is generally thought to be a constitutively active kinase [20]. Interestingly, the BMAL1 protein is hyperphosphorylated in CRY1/2-deficient mice [8,14], leading us to hypothesize that CRY proteins are involved in rhythmic BMAL1 modification. Here, we investigated the universal role and oscillatory mechanism of the circadian CK2-mediated BMAL1 phosphorylation. Accordingly, a novel role of CRY as a repressor for protein phosphorylation was found. We propose a model that explains how CRY proteins produce circadian oscillations and integrate posttranslational modification events (i.e., BMAL1 phosphorylation) in the negative limb of the core transcription–translation feedback loop.

## Results

### CK2-Mediated Circadian BMAL1-S90 Phosphorylation Regulates Mammalian Central and Peripheral Clocks

**Fig 1:**
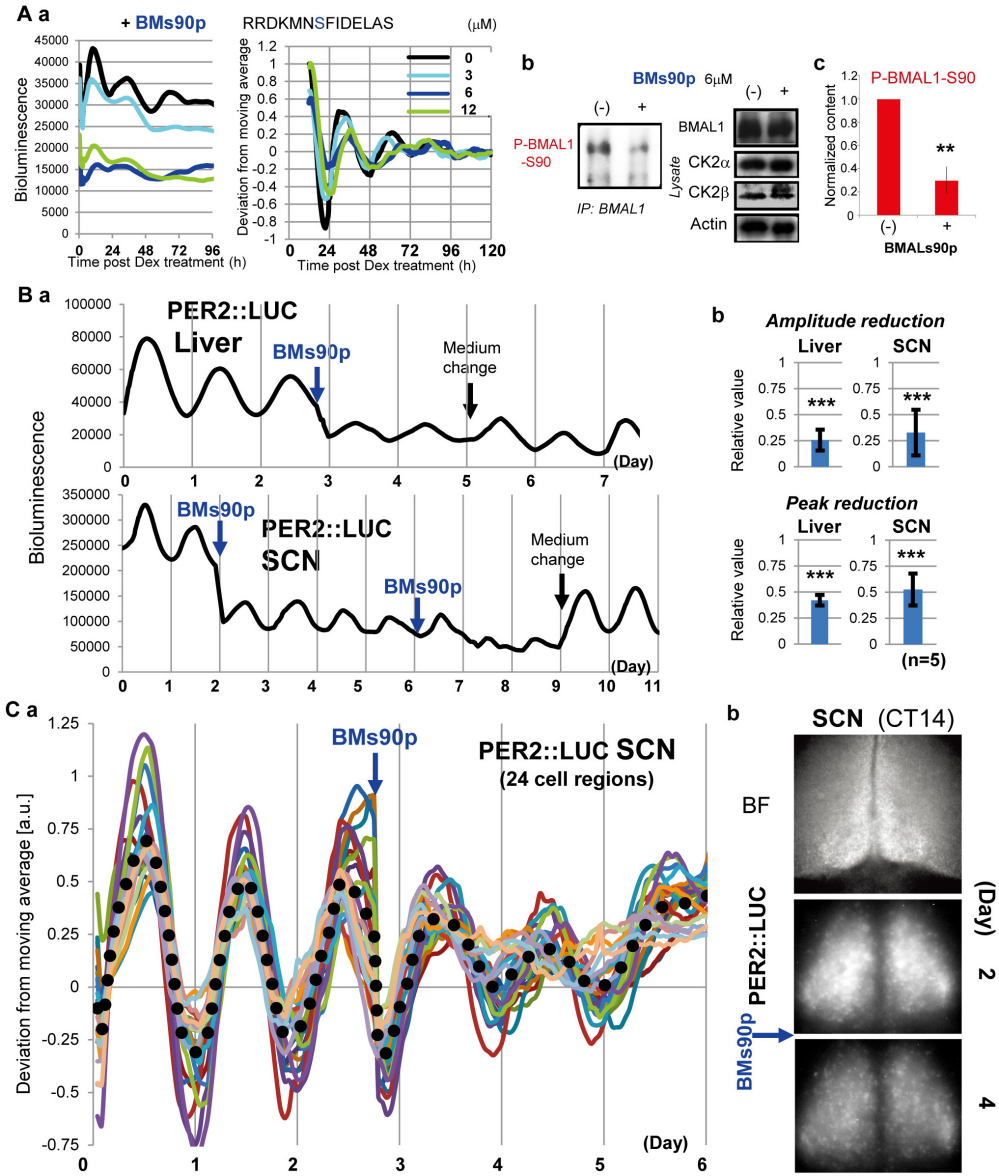
CK2-mediated circadian BMAL1-Ser90 phosphorylation regulates the central and peripheral clocks. (A) Clock performance and BMAL1 phosphorylation in NIH-3T3:Per2L cells after treatment with BMs90p (a 14 amino acid BMAL1 peptide containing S90; concentrations as indicated) for 30 min and subsequent clock-synchronization by dexamethasone (Dex) treatment for 30 min. (a) Cell cultures were monitored for luciferase activity by real-time bioluminescence assay. Representative raw (left) and detrended/averaged (right) profiles are shown (*n* = 4). (b) Immunoblot (IB) analysis of BMAL1-immunoprecipitates (IP) and lysates from BMs90p-treated cells for BMAL1, P-BMAL1-S90 (Ser90-phoshorylated BMAL1), CK2α, CK2β, and actin (control). Shown are representative images of triplicate experiments. (c) Quantification of P-BMAL1-S90 levels in BMs90p treated cells (*n* = 3). Values in untreated cells were set as 1. Error bars indicate standard deviation (SD). (B) Clock performance of organotypic slices of liver and SCN from *mPER2* ^*Luc*^ mice following treatment with 6 μM BMs90p or mock treatment with fresh medium around the PER2::LUC trough phase (liver; ~CT [Circadian Time] 5, SCN; ~CT2). (a) Luciferase activity was monitored by real-time bioluminescence imaging. Note the recovery of BMs90P dampened rhythms by medium change. (b) Quantification of rhythm amplitude and peak bioluminescence after BMs90p treatment (*n* = 5), in which values in untreated slices are set as 1. Error bars indicate SD. (C) Clock performance of small cell clusters in organotypic SCN slices from PER2::LUC mice following treatment with 6 μM BMs90p around the trough phase (~CT2). (a) Luciferase activity was monitored by real-time bioluminescence imaging (*n* = 24). Shown are detrended (colored lines) and averaged values (dotted line). (b) Representative examples of bright field (BF) and Per2L images at CT14 (around the peak phase) pre- and post-BMs90p treatment (day 2 and 4 respectively).

To investigate the critical role of CK2-mediated circadian phosphorylation of BMAL1 at Ser90 (referred to as BMAL1-S90) in regulating the central clock in the SCN and peripheral clocks in the liver, and in cultured fibroblasts, we designed a competitive inhibitor of BMAL1-S90 phosphorylation, consisting of a 14 amino acid BMAL1 peptide (BMs90p), centered around Ser90 (Fig 1Aa). As expected, and in line with S90A mutagenesis data [12], BMs90p dose-dependently (optimum at ~6 μM) suppressed both the formation of BMAL1 phosphorylated at Ser90 (hereafter referred to as P-BMAL1-S90), and *mPer2* promoter-driven luciferase (Per2L) bioluminescence rhythms in dexamethasone (Dex) clock-synchronized NIH-3T3 fibroblasts (Fig 1Aa, 1Ab and 1Ac). P-BMAL1-S90 was recovered only partially around 2-6h post-treatment (S1A Fig). In contrast, a control peptide with Ser90 replaced by Ala (BMa90p) did not inhibit Per2L rhythms and P-BMAL1-S90 phosphorylation, demonstrating the specificity of BMs90p (S1B Fig). Thus, BMs90p perturbs the circadian core oscillator (as evident from the suppressed Per2L rhythms) by inhibiting BMAL1-S90 phosphorylation. To test the effect on the central and peripheral clocks, we applied BMs90p to SCN and liver organotypic slices from *mPER2* ^*Luc*^ mice [21]. BMs90p-treatment provoked an evident reduction of the amplitude (liver; 0.255, SCN; 0.322; average value pre-treatment set as 1) and peak intensity (liver; 0.420, SCN; 0.525; average value pre-treatment set as 1) of Per2L (PER2::LUC) Per2L rhythms, without any evident phase-shifting effect (Fig 1B). Notably, this effect was only observed when BMs90p was administered at the trough of Per2L reporter gene activity (liver; ~CT5, SCN; ~CT2) (S2 and S3 Figs). Similar to the whole slice data, BMs90p suppressed Per2L rhythms in the SCN, as determined by imaging multiple (*n* = 24) small SCN cell clusters (Fig 1C, S4 Fig and S1 Movie). Taken together, these data strongly suggest a pivotal role of cyclic CK2-mediated BMAL1-S90 phosphorylation in the circadian oscillator of SCN neurons (central clock) and liver cells (peripheral clocks).

### CRY Proteins Inhibit BMAL1-S90 Phosphorylation

How are circadian oscillations in P-BMAL1-S90 levels generated? A clue may be found in previous observations that BMAL1 is constitutively hyperphosphorylated in CRY1/2-deficient (CRYdKO) cells with a dysfunctional clock [8,14].

**Fig 2:**
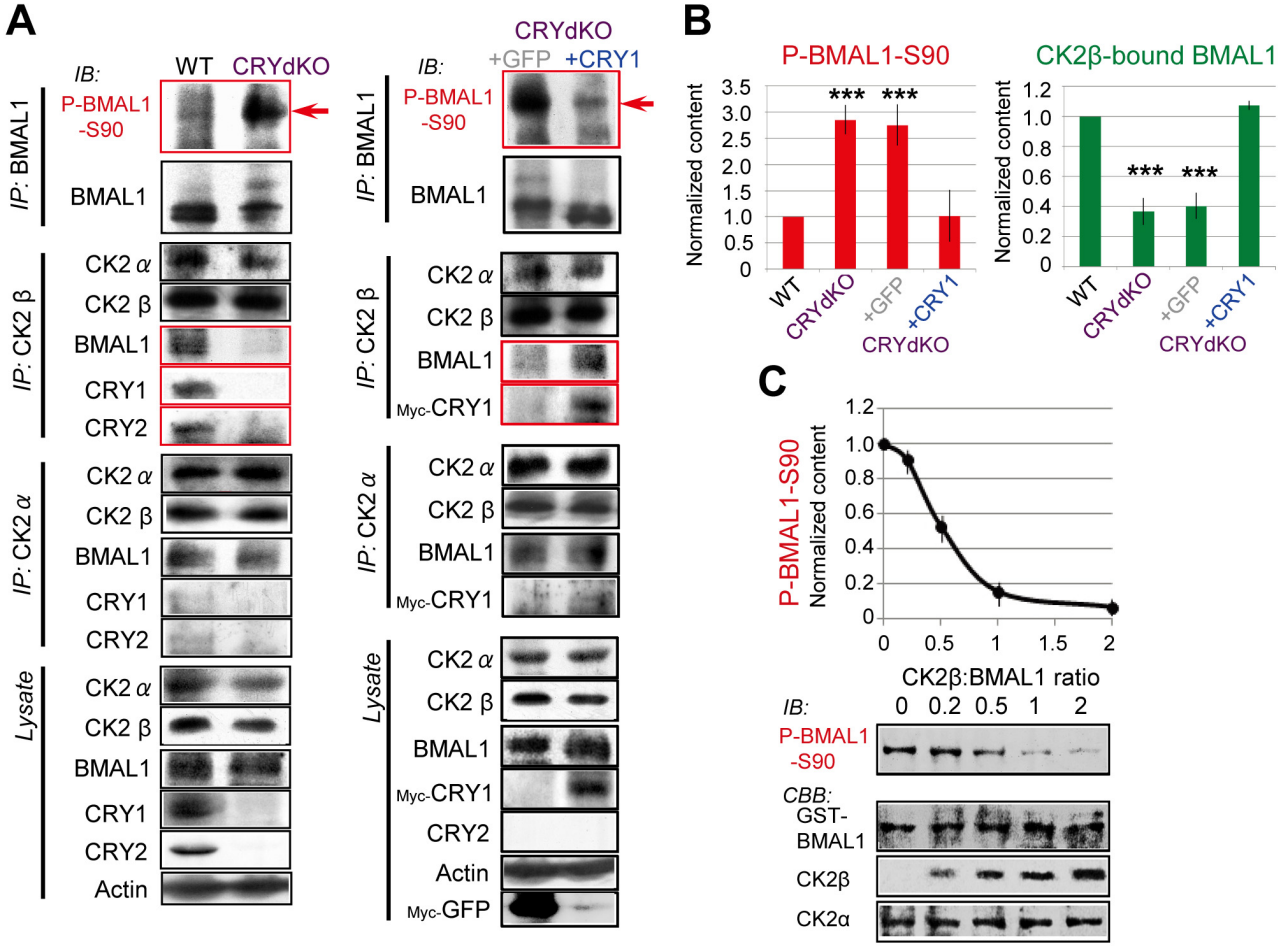
CRY regulates CK2-mediated BMAL1-Ser90 phosphorylation. (A) Co-immunoprecipitation experiments with non-clock synchronized wild type (WT) and CRYdKO (*Cry1* ^*-/-*^ */Cry2* ^-/-^) mouse embryonic fibroblasts (MEFs), and CRYdKO MEFs stably expressing *mCry1* promoter-driven Myc-CRY1 or CMV-promoter-driven Myc-GFP (control). IB analysis of BMAL1-, CK2α-, and CK2β-IP and lysates for BMAL1, P-BMAL1-S90, CK2α, CK2β, CRY1, CRY2, and actin (control). Shown are representative examples of *n* = 3 experiments. Red arrows indicate the position of major P-BMAL1-S90 (~90 kDa). (B) Quantification of P-BMAL1-S90 and CK2β–BMAL1 levels. Values in WT MEFs are set as 1. Error bars indicate SD. (C) In vitro analysis of CK2α-mediated BMAL1-S90 phosphorylation. Mixtures of CK2α and CK2β subunits and GST-BMAL1 were subjected to an in vitro kinase assay. BMAL1-Ser90 kinase activity was measured by IB analysis with anti-P-BMAL1-S90 (representative examples of *n* = 3 experiments), and plotted against CK2β/ (GST-) BMAL1 ratio. Values were normalized against activity in the absence of CK2β, which was set as 1. Error bars indicate SD. Note the inhibition of CK2α-mediated BMAL1-S90 phosphorylation by CK2β.

We therefore first investigated whether hyperphosphorylation of BMAL1 in CRYdKO cells includes Ser90. As shown in Fig 2A and 2B, P-BMAL1-S90 level was significantly higher in CRYdKO cells than in wild-type (WT) cells. Importantly, expression of *Cry1* promoter-driven Myc-CRY1 in CRYdKO cells (S5 Fig) caused the P-BMAL1-S90 level to return to WT levels (Fig 2A and 2B), suggesting that the CRY proteins act as suppressors of BMAL1-S90 phosphorylation.

Based on these results, we next focused on CK2-mediated BMAL1-S90 phosphorylation and the role of CRY proteins therein. Whereas the majority of CK2α catalytic subunits are likely recruited to a CK2β (regulatory unit) dimer to form a constitutively active CK2α_2_β_2_ tetramer that can phosphorylate a wide range of substrates [20], CK2β behaves as a strong inhibitor of CK2α-mediated BMAL1 phosphorylation in a dose-dependent manner [12]. Notably, CK2β does not directly inactivate CK2α [20]. Rather, CK2α activity is thought to fluctuate by the influence of yet unidentified cellular molecules.

As reported previously [12], we demonstrated that the CK2α monomer, but not CK2α2β2, phosphorylates BMAL1 at Ser90, and that CK2 kinase activity dramatically declined at a ratio of CK2β/CK2α ≥1 (Fig 2C). Similarly, we confirmed that CK2β inhibits CK2α-mediated BMAL1-S90 in vitro kinase activity as a function of the CK2β/BMAL1 ratio (Fig 2C). Kinase activity dramatically declined at a ratio of CK2β/BMAL1 ≥1, suggesting that CK2β interferes with CK2α monomer-mediated BMAL1-S90 phosphorylation by direct interaction with BMAL1. Indeed, in WT cell homogenates, CK2β is shown to co-precipitate with BMAL1 (Fig 2A). In marked contrast, however, BMAL1–CK2β interactions were significantly reduced in hyperphosphorylated P-BMAL1-S90 containing CRYdKO cells, while the amount of CK2α bound to BMAL1–CK2β complexes was comparable to that observed in WT cells (Fig 2A and 2B). Taken together, these data suggest that the amount of CK2α interacting with BMAL1 itself does not reflect the BMAL1 phosphorylation status. Rather, it points to a model in which CK2β is recruited to BMAL1 to inhibit CK2α activity. In the absence of BMAL1–CK2β interactions in CRYdKO cells, CRY proteins are likely candidates for such recruiting function.

We therefore assessed the binding ability of CK2 subunits to mammalian CRY1/2. In vitro, recombinant GST-tagged CK2α, α′ and β subunits could pull down CRY1 and CRY2 (S6A Fig). Thus, both CK2α and β subunits can bind to BMAL1, as well as to CRY1/2. Nonetheless, consistent with the data shown in Fig 2A, CRY proteins preferentially bind to CK2β. Notably, CRY1/2 can still interact with CK2β in BMAL1-deficient cells, demonstrating that BMAL1 is not required for CRY-CK2β interactions (S6B Fig). These results indicate that CRY proteins mediate BMAL1–CK2β binding by sequentially interacting with CK2β. Moreover, expression of Myc-CRY1 (resembling native CRY1 in that it also preferentially binds to CK2β; see Fig 2A) resets the level of BMAL1-bound CK2β to WT levels (Fig 2A and 2B). As the level of BMAL1-bound CK2α remained unchanged by Myc-CRY1 expression, we propose a model in which CK2α-mediated BMAL1-S90 phosphorylation is cyclically inhibited by CRY-dependent binding of CK2β to BMAL1, resulting in rhythmic P-BMAL1-S90 levels.

### CRY-Mediated BMAL1–CK2β Association Periodically Suppresses CK2α-Mediated BMAL1-S90 Phosphorylation to Produce Rhythmic Kinase

**Fig 3:**
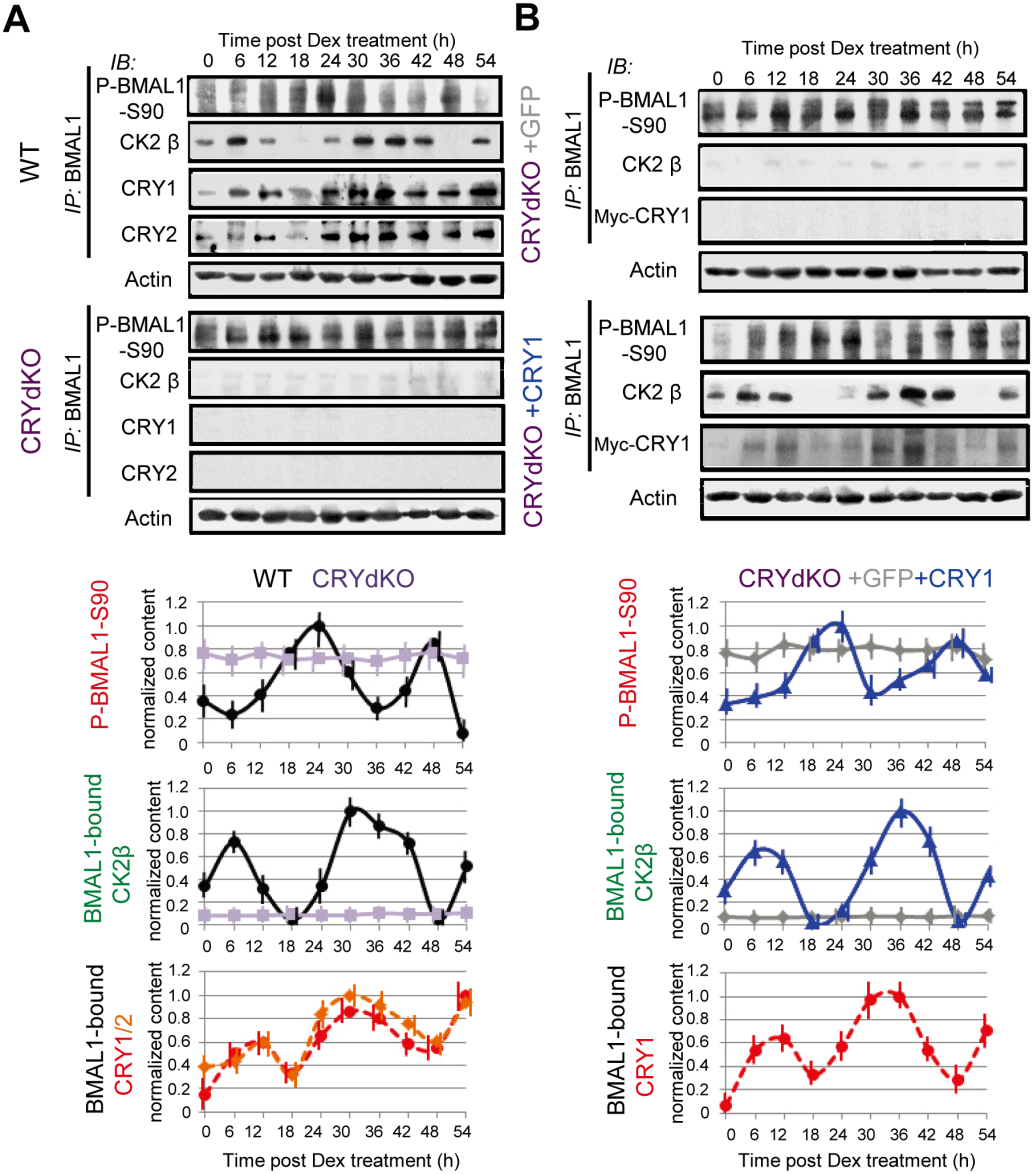
CRY is required for circadian CK2-mediated BMAL1-Ser90 phosphorylation, with inverse phase to BMAL1–CK2β binding rhythm. BMAL1 phosphorylation and CK2β, CRY1, and CRY2 interaction profiles in wild type (WT), CRYdKO (*Cry1* ^*-/-*^ */Cry2* ^-/-^) MEFs (A) and CRYdKO MEFs stably expressing *mCry1* promoter-driven Myc-CRY1 or CMV-promoter-driven Myc-GFP (control) (B). Cells were Dex-synchronized and harvested at 6 h intervals. BMAL1-IP and lysates were subjected to IB analysis for P-BMAL1-S90, CK2β, CRY1, CRY2 and actin (loading control). Shown are representative examples of *n* = 3 experiments (upper panels). P-BMAL1-S90 and BMAL1-bound CK2β, CRY1 and CRY2 levels were quantified and normalized against actin levels (lower panels). Maximum values in WT/CRYdKO+CRY1 were set as 1. Error bars indicate SD.

To test our hypothesis that the CRY-dependent periodic binding of CK2β to BMAL1 results in circadian P-BMAL1-S90 oscillation, we first examined the effects of CRY1/2 deficiency on the circadian pattern of P-BMAL1-S90 levels. As expected, and in contrast to the robust circadian oscillations in Dex-synchronized WT cells, P-BMAL1-S90 levels were constitutively expressed at high levels in CRYdKO cells (Fig 3 and S7 Fig). Moreover, periodic Myc-CRY1 expression (CRYdKO+CRY1) restored the circadian P-BMAL1-S90 oscillation, which peaked at a similar time (18–24 h after Dex treatment) as in WT cells (Fig 3B). The levels of BMAL1-bound CK2β in WT and Myc-CRY1 clock-rescued (CRYdKO+CRY1) cells exhibited robust circadian oscillation with peaks at 6–12 h and approximately 36 h with a phase nearly inverse to that of the P-BMAL1-S90 oscillation, whereas CRYdKO (with or without GFP) exhibited constitutively high P-BMAL1-S90 levels (Fig 3A and 3B). Reciprocally, CK2β-IP uncovered a similar temporal interaction pattern of BMAL1 with CK2β (S7 Fig). Moreover, CRY1 and CRY2 were shown to co-precipitate with BMAL1 or CK2β in a circadian manner and in phase with BMAL1–CK2β interactions (Fig 3A and S7 Fig). Notably, the circadian oscillation pattern of CK2β–CRY1/2 closely matched BMAL1–CK2β, rather than BMAL1–CRY1/2 rhythms (S7 Fig). As CK2β–CRY1/2 complexes can be formed in the absence of BMAL1 (S6B Fig), these data suggest that CRY proteins periodically facilitate BMAL1–CK2β interaction by first associating with CK2β. Moreover, circadian changes in posttranslational modification events may affect circadian patterns of BMAL1–CRY1/2–CK2β complexes. CK2α/β levels remained constant over time (S7 Fig), indicating that CK2α/β levels do not determine circadian P-BMAL1-S90 oscillation. These data lead us to conclude that the cyclic phosphorylation of BMAL1-S90 originates from periodic suppression of CK2α-mediated BMAL-S90 phosphorylation through CRY-mediated BMAL1–CK2β association.

### Live Cell Monitoring Reveals CRY-Enhanced Circadian Oscillation of BMAL1–CK2β Association

**Fig 4:**
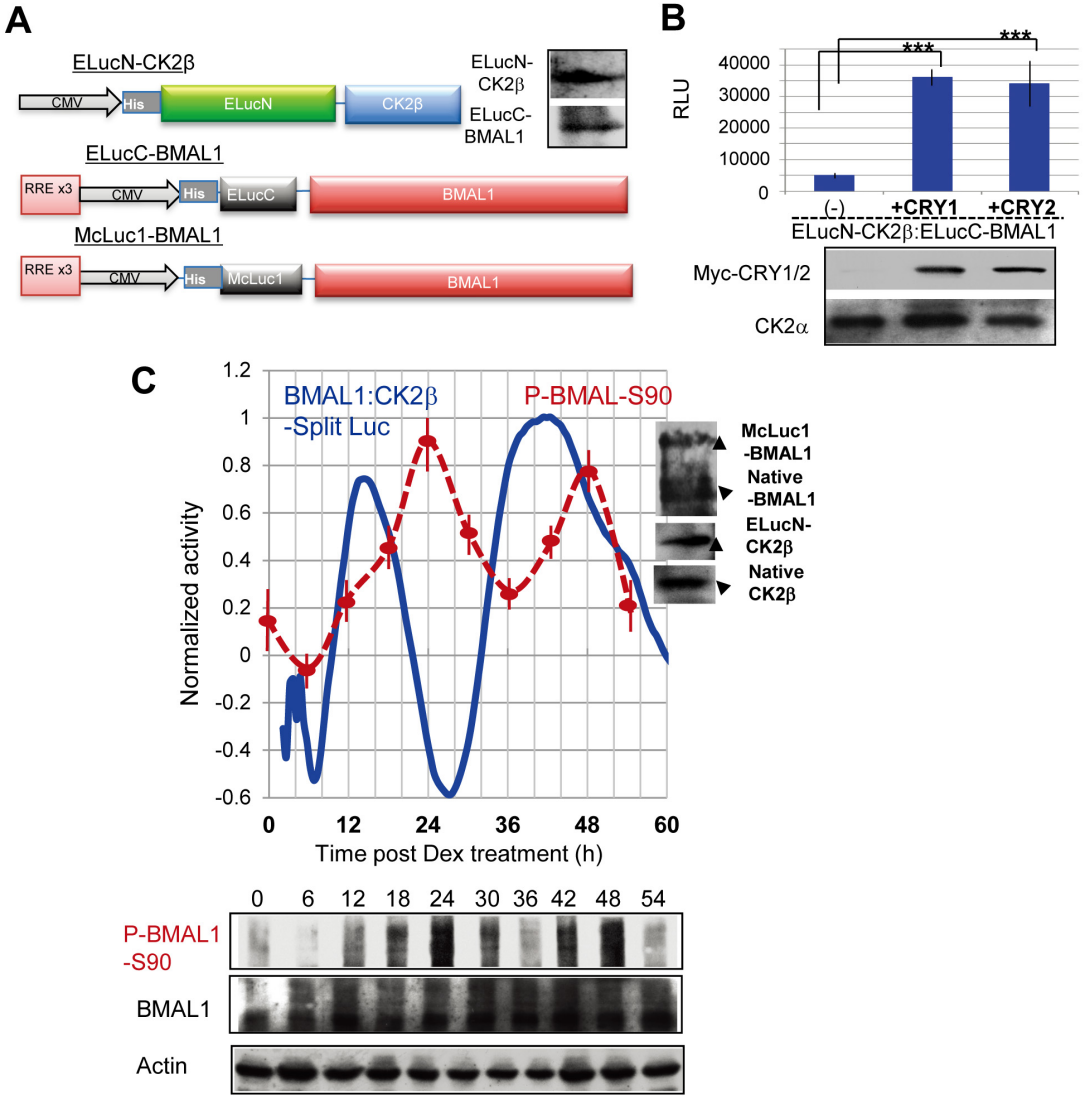
CRY-enhanced circadian oscillation of BMAL1–CK2β association in living cells. (A) Graphic representation of ELucN-CK2β, McLuc1-BMAL1, and ELucC-BMAL1 vectors for real-time bioluminescence imaging of CK2β-BMAL1 interactions as monitored by the Split Luc complementation system. IB analysis with anti-BMAL1 and CK2β antibodies reveals ectopic expression of His-tagged ELucC-BMAL1 (~100 kDa) and ELucN-CK2β ELucN-CK2β (~70 kDa) in Cos7 cells. (B) Cos7 cells were transfected with ELucN-CK2β and ELucC-BMAL1 (2.5 μg), and either Myc-HA-CRY1, Myc-CRY2, or (control) pcDNA (−) (5 μg). The presence of ectopically expressed CRY1/2 and native CK2α was confirmed by IB analysis (lower panel). BMAL1–CK2β binding was monitored by real-time bioluminescence imaging and plotted as the average value of peak bioluminescence levels (*n* = 5 experiments; upper panel). Error bars indicate SD. Note that BMAL1–CK2β binding was significantly facilitated by CRY1 and CRY/2. (C) Circadian rhythms in BMAL1–CK2β binding in Dex-synchronized U-2OS cells were monitored by real-time bioluminescence imaging of Split Luc activity (upper panel). The circadian profiles (Period = 25.3 h, Acrophase = 15.1 h) (*n* = 5; blue line) are shown with normalized values to the maximum value that was set as 1. Expression of McLuc1-BMAL1, native BMAL1, ELucN-CK2β, and native CK2β was confirmed by IB analysis (upper panel, insets). The circadian profile of P-BMAL1-S90 levels (representative examples of *n* = 3 experiments) was determined by IB analysis (lower panel) with anti-P-BMAL1-S90), quantified and normalized to actin content. Maximum values were set as 1 (upper panel: dotted red line). Error bars indicate SD.

To rule out the possibility of nonspecific associations in pull-down experiments, we next used a Split Luc complementation assay [22,23] for real-time monitoring of CK2β–BMAL1 interactions in living cells. In such an assay, bioluminescence can be detected only when N-(ELucN)- and C-(McLuc1 or ELucC)- tagged proteins associate and allow Luc moieties to complement each other and form active luciferase (Fig 4A). To this end, we ectopically expressed ELucN-CK2β and McLuc1/ELucC-BMAL1 in Cos7 and U-2OS cells at a level comparable to that of the native proteins (Fig 4A and 4C).

In Cos7 cells over-expressing Myc-tagged CRY1 or CRY2, bioluminescence due to BMAL1–CK2β interactions was significantly enhanced (approximately 6-fold; *p* < 0.001) (Fig 4B). This demonstrates that, consistent with the co-immunoprecipitation data obtained with CRYdKO-transfected MEFs (see Figs 2 and 3), BMAL1–CK2β binding in living cells requires CRY1/2. The in vitro CK2β-mediated inhibition of BMAL1-S90 phoshorylation (Fig 2C) and the BMAL1–CK2β binding in living cells (Fig 4B) strongly indicate that CRY proteins facilitate BMAL1–CK2β binding and subsequent suppression of CK2α-mediated BMAL1-S90 phosphorylation.

Dex-synchronized U-2OS cells exhibit robust circadian rhythmicity and can express high levels of ectopic proteins [24]. To investigate temporal changes in BMAL1–CK2β interactions, we monitored Split Luc activity in real-time mode in U-2OS cells and observed a robust circadian oscillation of BMAL1–CK2β binding, peaking approximately 15 h and 40 h after Dex-synchronization, and as such, inversely phased to P-BMAL1-S90 oscillations (Fig 4C and S8A and S8B Fig). These P-BMAL1-S90 patterns are consistent with those of asynchronous WT and CRY1-rescued CRYdKO MEFs (Fig 2A and 2B). As the RREx3/CMV promoter is constitutively active (see S8C Fig), the observed circadian BMAL1–CK2β Split Luc activity originates from rhythmic BMAL1-CK2β interaction rather than rhythmic BMAL1 expression. Ectopic expression of BMAL1 and CK2β in the Split Luc assay did not affect endogenous circadian phase or amplitude of the circadian core oscillator, as monitored through *Bmal1*-promoter driven luciferase activity (S9 Fig). Accordingly, the circadian patterns of BMAL1–CK2β association monitored by the Split Luc assay represent a nearly endogenous circadian pattern. Taken together with the result of Figs 3 and 4B, the antiphase circadian oscillations of BMAL1–CK2β interactions (as revealed by the binding Split Luc assay) and P-BMAL1-S90 rhythms strongly suggests that under physiological conditions (i.e., in the living cell) cyclic CRY-mediated BMAL1–CK2β association drives circadian BMAL1-S90 phosphorylation.

### CRY1 and BMAL1 Regions Critical for BMAL1–CK2β Association

**Fig 5:**
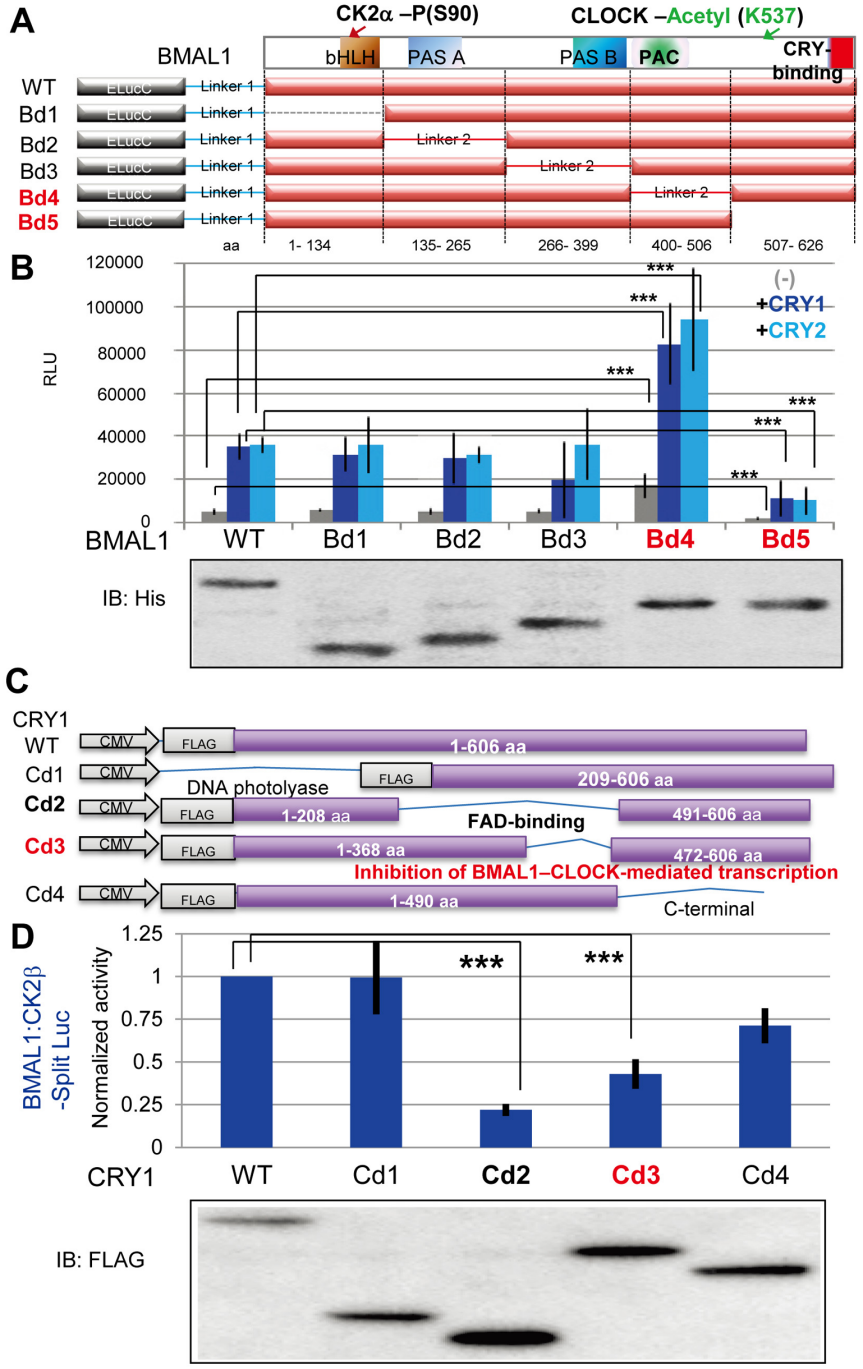
Identification of CRY1 and BMAL1 regions involved in CRY-enhanced BMAL1–CK2β binding. (A) Schematic representation of Split Luc mBMAL1 deletion mutant expression vectors. (B) Cos7 cells were transfected with ELucN-CK2β and either full length (WT) or mutant (Bd1-5) ELucC-BMAL1, in combination with either pcDNA (-; negative control), Myc-HA-CRY1, or Myc-CRY2. Expression of ELucC-BMAL1-full length and deletion mutants (Bd1-5) was confirmed by immunoblot analysis using anti-His antibody (lower panel). BMAL1–CK2β binding was monitored by a real-time bioluminescence imaging (upper panel). Shown are averaged peak bioluminescence values (*n* = 6 experiments). Error bars indicate SD. (C) Schematic representation of mCRY1 deletion mutant expression vectors. (D) Cos7 cells were transfected with ELucN-CK2β and ELucC-BMAL1, in combination with either full length (WT) or mutant (Cd1-4) pcDNA-FLAG-CRY1. Expression of CRY1-full length and deletion mutants (Cd1-4) was confirmed by immunoblot analysis using FLAG antibody (lower panel). BMAL1–CK2β binding was monitored by a real-time bioluminescence imaging (upper panel). Shown are averaged peak bioluminescence values (*n* = 6 experiments). Error bars indicate SD.

Next, we generated a panel of ELucC-mBMAL1 deletion constructs (Fig 5A) to determine the region of BMAL1 [25] critical for BMAL1–CK2β interaction. BMAL1–CK2β binding was detected at comparable levels in Cos7 cells expressing ELucN-CK2β with either ELucC-BMAL1-WT (full length) or deletion mutants in the N-terminal half of BMAL1 (Bd1-3), and was stimulated by co-expression of CRY1 or CRY2 (Fig 5B). However, irrespective of the presence of CRY1/2, expression of a BMAL1 mutant protein lacking the CRY-binding domain (Bd5) [26,27] resulted in significantly lower levels of bioluminescence (approximately 30%; *p* < 0.001) as compared to BMAL1-WT (Fig 5B), indicating that the C-terminal region of BMAL1 is critical for BMAL1–CK2β binding. Yet, BMAL1-Bd5:CK2β Split-Luc activities were significantly enhanced by co-expression of CRY1 (*p* < 0.001) or CRY2 (*p* < 0.001), suggesting that CRY binding to CK2β can also enhance CK2β binding to BMAL1 without direct physical interaction between CRY and BMAL1. Moreover, independent of the presence of CRY1/2, expression of the Bd4 mutant lacking the BMAL1 PAC (PAS-associated C-terminal) domain resulted in significantly higher levels of bioluminescence (approximately 2-fold; *p* < 0.001) as compared to BMAL1-WT (Fig 5B), implicating this region as a potential regulatory site for BMAL1–CK2β binding. CK2β binding with WT BMAL1, Bd4, and Bd5 was largely enhanced by ectopic CRY1/2 expression. These findings identified critical regions in BMAL1 for CRY-enhanced binding to CKβ.

To identify CRY1 protein regions critical for facilitating BMAL1–CK2β interaction, we generated a panel of mCRY1 deletion constructs (Fig 5C) for co-expression with ELucN-CK2β and ELucC-BMAL1 in Cos7 cells. CRY-facilitated BMAL1–CK2β binding, as detected by the Split-Luc assay, was not significantly altered by deletion of either the N-terminal DNA photolyase domain (Cd1) or the C-terminal region (Cd4) of CRY1 (Fig 5D). However, co-expression of a CRY1 mutant protein lacking the FAD-binding domain (Cd2) [28] resulted in significantly lower levels of bioluminescence (approximately 20%; *p* < 0.001) as compared to CRY1-WT (Fig 5D), indicating that the FAD-binding domain of CRY1 is critical in enhancing BMAL1–CK2β binding. Notably, co-expression of a mutant CRY1 protein (Cd3), lacking the pivotal region for inhibition of BMAL1–CLOCK-mediated transcription in the FAD-binding domain [28,29] also resulted in significantly lower levels of bioluminescence (approximately 40%; *p* < 0.001) as compared to CRY1-WT (Fig 5D). This finding indicates that the FAD-binding region is not only critical for suppression of CLOCK–BMAL1 -mediated transcription activation but also for CK2α-mediated BMAL1-S90 phosphorylation by facilitating BMAL1–CK2β binding.

### BMAL1-K537 Acetylation Reduces BMAL1-S90 Phosphorylation via Enhancing BMAL1–CK2β Association

BMAL1-K537 acetylation has been shown to facilitate the recruitment of CRY1/2 to BMAL1 [4]. To examine whether BMAL1-K537 acetylation affects S90 phosphorylation and CRY-mediated BMAL1–CK2β binding, we first stably transfected *Bmal1* ^*-/-*^ knockout MEFs with a *mBmal1* promoter-driven wild type (Myc-BMAL1-WT) or acetylation mutant BMAL1 (Myc-BMAL1-K537R) expression construct. BMAL1-K537R retains the ability for S90 phosphorylation (S10 Fig), indicating that K537 acetylation is not prerequisite for S90 phosphorylation. S90 phosphorylation levels were even higher for BMAL1-K537R than for BMAL1-WT. Interestingly, CK2β and CRY1/2 binding to BMAL1-K537R was reduced as compared to BMAL1-WT (S10 Fig), suggesting that K537 acetylation is required for CRY-mediated recruitment of CK2β to BMAL1.

**Fig 6:**
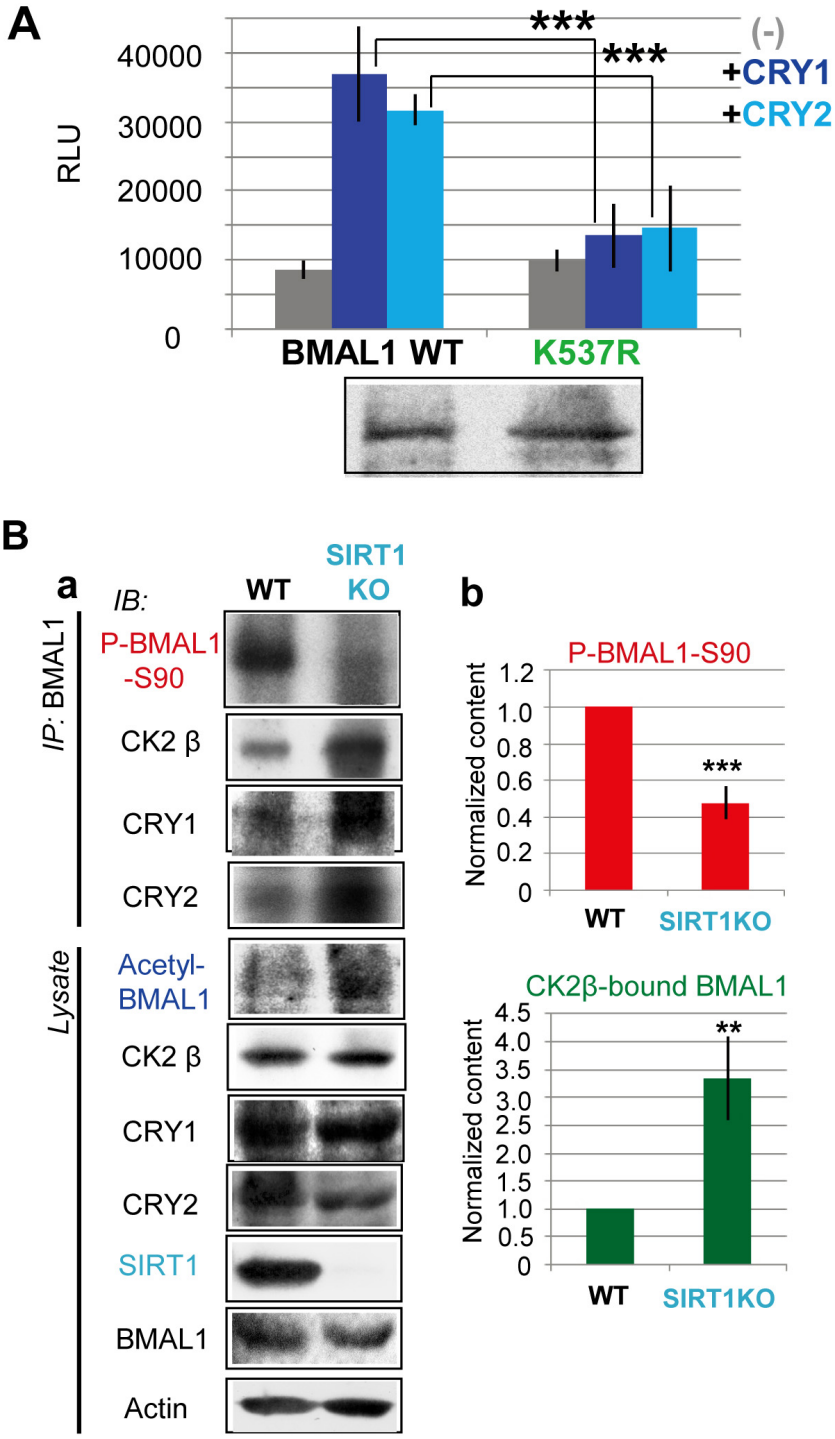
BMAL1-K537 acetylation reduces BMAL1-S90 phosphorylation via enhancing BMAL1–CK2β association. (A) Reduced CRY-enhanced CK2β binding to mutant BMAL1-K537R, lacking a CLOCK-mediated acetylation site. As in Fig 5, except that an acetylation mutant ELucC-BMAL1 vector (K537R) was used. (B) Reduction of CK2-mediated BMAL1-S90 phosphorylation in SIRT1KO (*Sirt1* ^*-/-*^) cells, lacking the major clock-regulating deacetylase SIRT1. (a) Co-immunoprecipitation experiments with WT and SIRT1KO MEFs 23h after Dex treatment. IB analysis of BMAL1-IP and lysates for P-BMAL1-S90, CK2β, acetyl-BMAL1, SIRT1, BMAL1, and actin. Shown are representative examples of *n* = 3 experiments. (b) Quantification of P-BMAL1-S90 and BMAL1-bound CK2 β levels. Values in WT MEFs are set as 1. Error bars indicate SD.

Next, we performed a Split-Luc live cell assay experiment using an ElucC-mBMAL1-K537R mutant protein. In the absence of CRY1/2, the BMAL1-K537R–CK2β binding was not significantly different from BMAL1–CK2β binding. However, in the presence of CRY proteins BMAL1–CK2β interactions increased by approximately 4-fold, while BMAL1-K537R–CK2β binding did not significantly increase (*p* < 0.001 in comparison with WT) (Fig 6A). Thus, CLOCK-mediated acetylation of K537 in the C-terminal region of BMAL1 appears critical for CRY-enhanced BMAL1–CK2β binding.

To further investigate whether BMAL1-K537 acetylation up-regulates BMAL1–CK2β binding and subsequently represses BMAL1-S90 phosphorylation, we utilized a mouse fibroblast cell line deficient in silent information regulator 1 (SIRT1), a member of the sirtuin family of NAD+-dependent histone deacetylases (HDACs) that also targets acetyl BMAL1-K537 [30]. As previously reported [30], the acetyl-BMAL1-K537 level was substantially higher in SIRT1KO (*Sirt1* ^-/-^) MEFs than in WT MEFs (Fig 6Ba). Importantly, as predicted, P-BMAL1-S90 levels were significantly reduced in SIRT1KO MEFs (approximately 50% of control; *p* < 0.001) as compared to WT cells (Fig 6Bb). Consistently, BMAL1–CK2β binding was significantly increased (approximately 330% of control; *p* < 0.01) in SIRT1KO MEFs (Fig 6Bb). Consistent with our previous report [4,30], BMAL1–CRY1/2 binding was increased in SIRT1KO MEFs (Fig 6Bb).

Taken together, these data demonstrate that acetylation of BMAL1 at Lys537 facilitates BMAL1–CK2β association, and as such represses BMAL1-S90 phosphorylation.

### BMAL1-S90 Phosphorylation Is Prerequisite for BMAL1-K537 Acetylation and Subsequent Recruitment of CRY to BMAL1

For a variety of proteins phosphorylation has been shown to trigger subsequent acetylation events [31,32]. Accordingly, S90 phosphorylation is potentially involved in the regulation of BMAL1 acetylation. To assess the link between BMAL1-S90 phosphorylation and BMAL1-K537 acetylation and to further establish the integral role of the CK2-mediated BMAL1-S90 phosphorylation in the circadian core oscillator, we examined the effect of BMAL1-S90 mutation on BMAL1-K537 acetylation, which, as shown above, is, critical for CRY-mediated BMAL1–CK2β binding and P-BMAL1-S90 rhythms.

**Fig 7:**
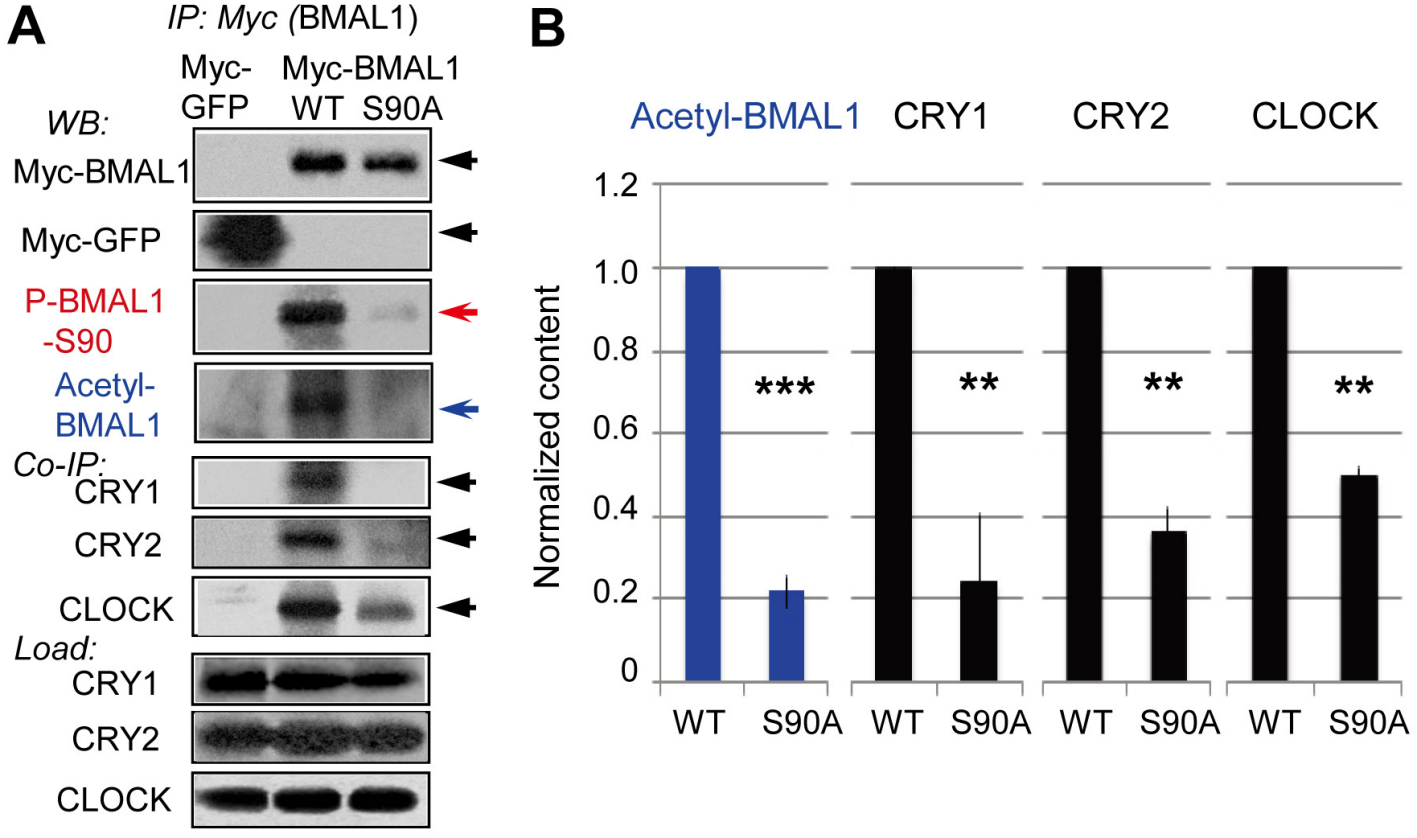
BMAL1-S90 phosphorylation is prerequisite for BMAL1-K537 acetylation and subsequent recruitment of CRY to BMAL1. (A) *Bmal1* ^−/−^ MEFs stably expressing *mBmal1* promoter-driven Myc-BMAL1-WT (wild type), Myc-BMAL1-S90A (CK2−phosphorylation site-deficient mutant), and CMV-promoter-driven Myc-GFP (control) were Dex-synchronized. The supernatants from the cells harvested 22 h after treatment were subjected to Myc-BMAL1 IP. The Myc-BMAL1 IPs and total lysates (Load) were subjected to IB analysis for Acetyl-BMAL1 and BMAL1- co-immunoprecipitated (Co-IP) CRY1/2 and CLOCK. (B) Normalized Acetyl-BMAL1 and BMAL1-bound CRY1/2 and CLOCK levels are shown in the graph (*n* = 3 experiments) and error bars indicate SD.

To this end, we stably expressed wild-type BMAL1 (BMAL1-WT), mutant BMAL1-S90A (with Ser90, located in the basic helix-loop-helix DNA binding motif, substituted for Ala) or GFP (negative control) in MEFs derived from clock-deficient *Bmal1*-null mice [7]. As shown in Fig 7A, Myc-BMAL1-WT and Myc-BMAL1-S90A were expressed at equal level. The acetylation level of BMAL1-S90A was dramatically reduced as compared to BMAL1-WT (approximately 20% of control; *p* < 0.001; Fig 7A and 7B). Thus, CK2α-mediated BMAL1-S90 phosphorylation might be a prerequisite for CLOCK-mediated BMAL1 K537 acetylation. Consistent with this notion, BMAL1–CLOCK levels were decreased (approximately 50% of control; *p* < 0.002) by the S90A mutation (Fig 7A and 7B), suggesting that reduction in BMAL1–CLOCK association is the cause of the reduced BMAL1-K537 acetylation level.

Furthermore, the BMAL1–CRY1/2 interactions were also significantly decreased by the S90A mutation (Fig 7A and 7B), indicating that S90 phosphorylation is prerequisite for K537-facilitated CRY recruitment to BMAL1. As previously reported, reduction of BMAL1–CRY interactions by K537R mutation demonstrates K537-acetylation-dependent recruitment of CRY1/2 to BMAL1 [4]. Thus, CK2α-mediated BMAL1-S90 phosphorylation is a prerequisite for CLOCK-mediated BMAL1-K537 acetylation, subsequent CRY1/2 recruitment to BMAL1 and CRY1/2-facilitated BMAL1–CK2β binding to regulate negative feedback suppression of clock gene expression and CK2α-mediated BMAL1-S90 phosphorylation, respectively. Our results do not exclude the existence of other BMAL1 phosphorylations, which directly trigger BMAL1-acetylation and other modifications, located downstream of CK2-mediated BMAL1-S90 phosphorylation.

## Discussion

Circadian BMAL1-S90 phosphorylation has been shown to be an important regulatory step in the mammalian core clock oscillator [12]. In the present study, we addressed the underlying mechanism and uncovered a vital interplay between CRY proteins and circadian BMAL1 phosphorylation. First, by applying a novel clock-perturbing peptide (BMs90p) to SCN and liver organotypic slices from *mPER2* ^*Luc*^ mice and subsequent live monitoring of circadian clock performance, we further highlighted a universal critical role of BMAL1-S90 phosphorylation in central and peripheral clocks. BMs90p (a small 14 amino acid peptide containing the BMAL1-Ser90 phosphorylation site targeted by CK2) behaves as a competitive inhibitor of BMAL1-S90 phosphorylation and was shown to reversibly blunt Per2L bioluminescence rhythms in a dose- and circadian time-dependent manner

Next, triggered by the observation that a CRY1/2-deficiency causes hyper-phosphorylation of BMAL1 [8,14], we focused on the molecular mechanism underlying circadian BMAL1-S90 phosphorylation and showed that in wild-type cells, circadian phosphorylation of BMAL1-S90 is accompanied by inverse phase cyclic association of BMAL1 with CK2β, a known inhibitor of CK2α-mediated BMAL1 phosphorylation [12]. Notably, a CRY1/2-deficiency abolishes BMAL1-CK2β interactions, and as such prevents cyclic inhibition of BMAL1-S90 phosphorylation, resulting in constitutively hyperphosphorylated BMAL1. P-BMAL1-S90 in CRY1/2 deficient cells could be rescued by rhythmic *Cry1* expression, which points to a model in which CRY proteins cyclically recruit CK2β to BMAL1 to inhibit CK2α activity.

To provide further evidence for this model, we developed a Split-Luc–based assay system for real-time monitoring of clock protein–protein interactions in living cells. Using this assay, we have shown that BMAL1 cyclically binds to CK2β and that circadian BMAL1–CK2β binding is enhanced by CRY proteins. Moreover, using the same Split-Luc approach in combination with mutant versions of the BMAL1 protein, we have shown that the PAC and CRY-binding domains in the C-terminal region of BMAL1, as well as BMAL1-K537 acetylation (known to enhance CRY-recruitment to BMAL1 [4]) are important in regulating BMAL1–CK2βbinding. Indeed, using SIRT1KO cells, we demonstrated that BMAL1-K537 hyper-acetylation reduces BMAL1-S90 phosphorylation through enhanced CRY-driven BMAL1–CK2β association. As BMAL1-S90 phosphorylation is prerequisite for BMAL1-K537 acetylation (see below), the low but significant P-BMAL1-S90 level in SIRT1KO MEFs is apparently sufficient to trigger BMAL1-K537 acetylation (Fig 6B). Reciprocally, BMAL1-S90A expressing MEFs, lacking BMAL1-S90 phosphorylation, cannot trigger significant BMAL1-K537 acetylation (Fig 7A and 7B).

By BMAL1-S90A mutagenesis, we showed that BMAL1-S90 phosphorylation is prerequisite for BMAL1-K537 acetylation. The S90A mutation significantly reduces the nuclear BMAL1–CLOCK levels [12,33] and S90-phosphorylated BMAL1 is mostly detected in the nuclear fraction (S11 Fig), strongly suggesting BMAL1 enters the nucleus promptly after S90 phosphorylation. Taken together with the lower K537 acetylation and CLOCK binding capacity of BMAL1-S90A, as compared to BMAL1-WT, it is assumed that K537 acetylation mainly occurs after BMAL1–CLOCK nuclear entry. The enhanced K537 acetylated/S90-phosphorylated BMAL1 level in *Sirt1* knockout cells suggests a mutual regulatory loop between K537 acetylation and S90 phosphorylation and supports the notion that S90 phosphorylation is prerequisite for K537 phosphorylation, while K537 acetylation represses S90 phosphorylation.

**Fig 8:**
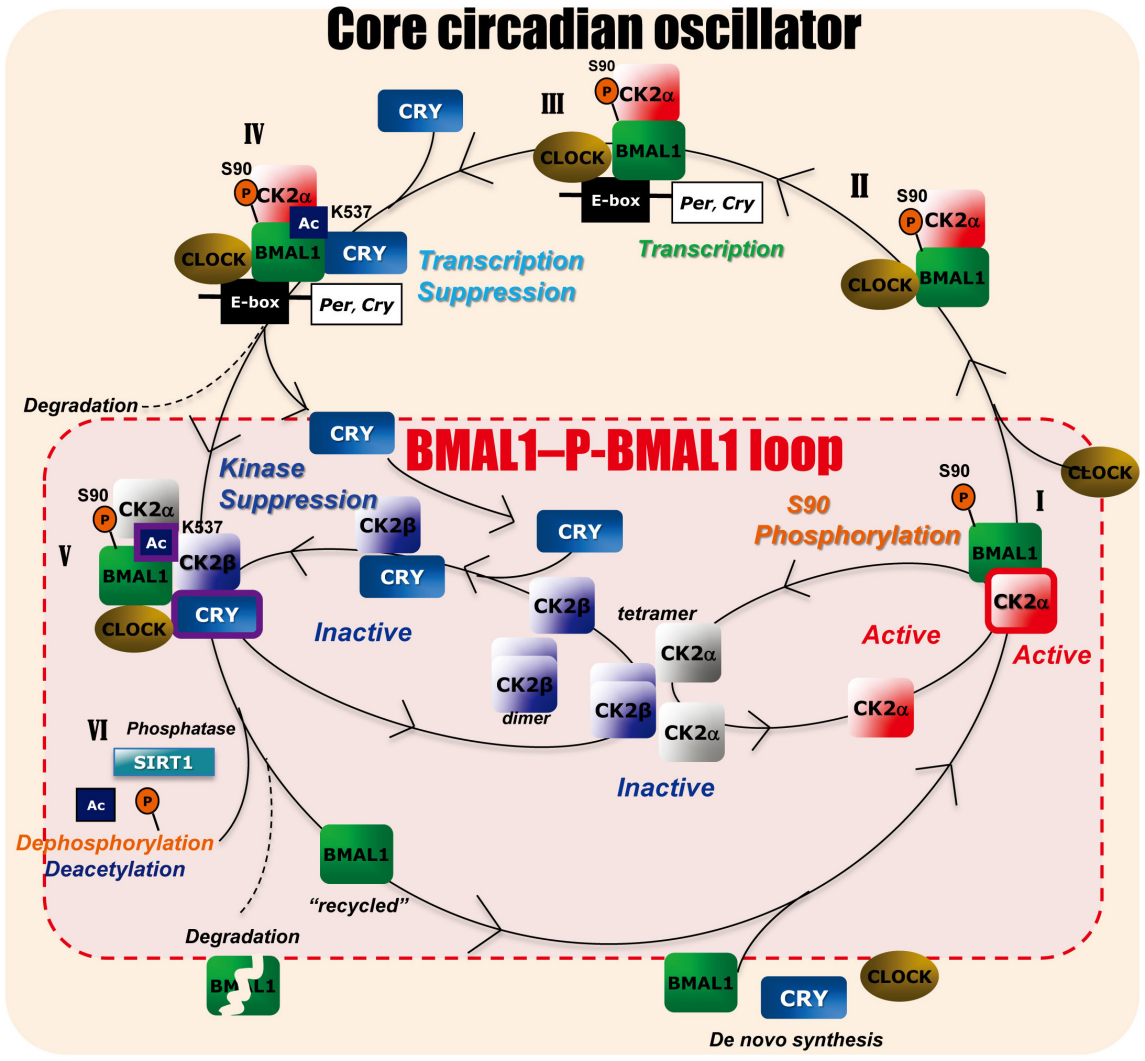
A CRY-based dual negative feedback model integrating the BMAL1– P-BMAL1 loop with the circadian core oscillator. Proposed model for CRY-CK2β–mediated circadian rhythms in BMAL1 phosphorylation by CK2α (the BMAL1–P-BMAL1 loop) as an integral part of the circadian core oscillator. In short, upon phosphorylation of BMAL1-S90 by CK2α (**step I**), BMAL1 binds to CLOCK (**step II**) to form a transcriptional activator complex for transcription of E-box promoter containing clock genes (i.e., *Cry* and *Per* genes) and clock-controlled genes (**step III**). CK2α remains bound to BMAL1 in a catalytically active state (as indicated by the red color). After a delay, CRY proteins will bind to the CLOCK–BMAL1–CK2α complex to inhibit E-box gene transcription (**step IV**). Upon dissociation of the BMAL1–CLOCK–CRY–CK2α complex from the DNA, CRY is released from the complex to allow CRY–CK2β binding, and subsequent BMAL1–CK2β binding, resulting in the formation of BMAL1–CLOCK–CRY–CK2β–CK2α complex (**step V**). This step, triggered by acetylation of BMAL-K537, renders CK2α inactive (as indicated by the grey color). After dissociation of the BMAL1–CLOCK–CRY–CK2β–CK2α complex, BMAL1 is degraded and/or dephosphorylated and deacetylated (**step VI**) to start a new cycle. For a detailed description of the model, see the Discussion section. For simplicity, PER proteins have not been included in the model and CRY1 and CRY2 proteins are collectively shown as CRY.

In conclusion, we established a circadian clock-controlling role of CK2 kinase, formerly thought to be a constitutively active kinase [20] in BMAL1 phosphorylation and uncovered a novel role of CRY as a regulator of cyclic CK2-mediated BMAL1 phosphorylation. Fig 8 illustrates our model for the molecular mechanism of the CK2-mediated posttranslational loop and its role in regulating the intracellular circadian core oscillator. In this model, cyclic CK2α-mediated BMAL1-S90 phosphorylation serves as the periodic gateway that controls BMAL1–CLOCK heterodimerization (step I) and time-delayed nuclear accumulation of BMAL1–CLOCK (step II) [12]. Step I and II may play a critical role in the events described below and serve as a time-delay factor that fine-tunes the circadian periodicity [14]. Therefore, we refer to CK2-mediated BMAL1-S90 phosphorylation as the first gate, probably located at the boundary between the cytoplasm and nucleus. Consistently, constitutive nuclear predominant BMAL1 localization in CRYdKO MEFs through the circadian cycle might be largely due to constitutive active BMAL1-S90 phosphorylation [14]. After BMAL1–CLOCK accumulates in the nucleus, E-box promoter containing clock genes, including *CRY1/2*, are temporally transactivated (step III). This is followed by negative feedback suppression of BMAL1–CLOCK transcription of E-box genes by the recruitment of CRY1/2 to BMAL1 (step IV), which is regulated by CLOCK-mediated BMAL1-K537 acetylation [4] and requires phosphorylated BMAL1-S90. In the next step (step V), because of the delayed surge in CRY1/2–CK2β binding, the BMAL1–CLOCK–CRY complex is released from the E-box. Thereafter, we hypothesize that CRY proteins are released from the complex to make way for newly incoming CRY1/2–CK2β complexes that bind to BMAL1–CLOCK via direct CRY–BMAL1 interaction. Deletion of the CRY-binding domain in BMAL1 does not completely abolish CRY-mediated enhancement of BMAL1–CK2β binding in the Split Luc assay (Fig 5), suggesting direct BMAL1–CRY interaction is not absolutely necessary for the enhancement of BMAL1–CK2β binding. Given that CRY has also been shown to bind to CLOCK [29], docking of CRY–CK2β to BMAL1–CLOCK may also involve CRY–CLOCK interactions. Through formation of CRY1/2–CK2β intermediates, CRY1/2 facilitates BMAL1–CK2β association. Notably, the release of CRY from and re-entry of CRY–CK2β in the BMAL1–CLOCK complex (instigated by the observation that CRY can bind CK2β in the absence of BMAL1) is the most speculative step in the model. In the absence of experimental/mechanistic evidence we cannot fully exclude that CRY proteins enhance BMAL1–CK2β binding while still bound to the BMAL1–CLOCK–CRY complex.

In vitro, CK2β can bind to BMAL1 in the absence of CRY and inhibit BMAL1-S90 phosphorylation by CK2α (Fig 2C and S6A Fig). Under these non-physiological conditions, inhibition of S90 phosphorylation may occur through (simultaneous) formation of CK2β-BMAL1 complexes that prevent CK2α from binding to BMAL1, and/or formation CK2α2β2 tetramers [20], which are probably incapable of phosphorylating BMAL1 [12]. However, as formation of a CK2α2β2 tetramer requires formation of a CK2β dimer that subsequently binds two CK2α monomers, and as in vitro BMAL1-S90 phosphorylation by CK2α is maximally inhibited at a CK2β:BMAL1 ratio of 1 (Fig 2C), interaction of 1 CK2β monomer rather than a tetramer with 1 BMAL1 molecule appears sufficient to inhibit S90 phosphorylation. In vivo, BMAL-CK2β association appears to block (BMAL1-bound) CK2α activity, rather than BMAL1–CK2α association, and requires the help of CRY proteins. Although we do not exclude a model in which CK2β is included in the BMAL1–CLOCK–CRY–CK2β–CK2α as a dimer or α2β2 tetramer complex, we consider inhibition of CK2α-mediated BMAL1-S90 phosphorylation by a CK2β monomer the most plausible option (step V). Taken together, inverse-phased circadian BMAL1–CLOCK–CRY–CK2β−CK2α complex formation might be the primary determinant for circadian CK2α-mediated BMAL1-S90 kinase activity. Next, S90 phosphorylated BMAL1 undergoes a SIRT1-mediated deacetylation step (step VI) [30] that likely liberates BMAL1 from the complex. Subsequent SUMOylation [3] and ubiquitination [34] of BMAL1 may target the protein for proteasomal degradation. In addition, non-degraded BMAL-S90 needs to be dephosphorylated by yet unknown phosphatases to initiate a new cycle through CK2α-mediated phosphorylation of BMAL1.

BMAL1-S90 phosphorylation by the CK2α monomer most likely occurs at step I (Fig 8). S90-phosphorylation of BMAL1 takes place in the cytoplasm and triggers CLOCK binding and subsequent BMAL1–CLOCK nuclear accumulation [12]. We have shown that BMAL1–CK2α complexes exist throughout the circadian cycle (S7 Fig). This suggests that the CK2α monomer remains bound to the BMAL1–CLOCK complex up to step IV. Likely, CK2α remains catalytically active (though its substrate is no longer available) and only gets inactivated after CRY-mediated binding of CK2β (step V).

In this study, we have unveiled the underlying mechanism for the cyclic CK2-mediated BMAL1 phosphorylation as a critical event in the mammalian circadian core clock machinery. BMAL1–P-BMAL1 loop forms a distinct interlocked loop in the clock machinery (step I, V, and VI) and have integral roles in the core circadian oscillator through periodic CRY-mediated negative feedback suppression. In this scenario, CRY proteins have a dual function. Strikingly, in addition to their known function as repressors of BMAL1–CLOCK-driven transcription, we found a novel role of CRY proteins as a repressor of CK2 protein kinase activity toward BMAL1-S90. Notably, we observed that the FAD binding region of CRY1, known to be essential for repression of BMAL1–CLOCK-driven transcription [28], is also critical for inhibition of CK2α-mediated BMAL1-S90 phosphorylation.

Interestingly, CLOCK-mediated BMAL1-K537 acetylation [4], through sequential recruitment of CRYs and then CRY1/2–CK2β to the BMAL1–CLOCK complex, acts as a common molecular key for evoking CRY-mediated feedback inhibition of BMAL1–CLOCK transcription activity and CRY-dependent suppression of BMAL1 phosphorylation. Ultimate verification of our model ideally requires in vitro reconstitution of the CRY-driven circadian BMAL1–P-BMAL1 loop, as shown for cyanobacterial KaiC phosphorylation [35]. In a first experiment in which purified recombinant CRY1 was added to the in vitro BMAL1-S90 phosphorylation assay (as performed in Fig 2C), we observed that despite its ability to bind BMAL1 (S12A Fig), CRY1 could not inhibit CK2α-mediated BMAL1-S90 phosphorylation (S12B Fig). This apparent difference markedly contrasts with the in vivo data, where CRY has been shown pivotal for CK2β-mediated inhibition of BMAL1-S90 phosphorylation by CK2α. Clearly, in vitro assays differ from the in vivo situation in that they do not take into account the effect of subcellular localization of the proteins studied, their interaction with DNA or chromatin, or the involvement of other protein partners. Moreover, in vitro synthesized proteins probably do not undergo posttranslational modification, leading us to hypothesize that CRY can only recruit CK2β to BMAL1 after acetylation of BMAL1-K537.

CK2 phosphorylates a large array of cellular proteins and is widely involved in regulating mammalian physiology [17]. However, temporal aspects of CK2 function are still elusive. Therefore, in addition to its role in the core clock, future investigations should focus on CK2-mediated circadian signaling as a regulator of various physiological and pathological pathways. A genome-wide phospho-proteomics study of periodic signaling systems focused on CK2 may help elucidate the chronobiological attributes of diverse physiological events and facilitate the development of therapies for circadian-system–related disorders [36], such as metabolic syndromes, cancer, and neuropsychiatric diseases. Recently, we demonstrated that CK2-BMAL1 kinase plays a critical role in controlling protective pathways evoked by reactive oxygen species and is crucial for preventing oxidative-stress–related diseases [37].

## Materials and Methods

### Biochemical Analysis

Immunoprecipitation and immunoblotting were performed using sample solutions as previously described [4,12,14]. Antibodies utilized in the immuno-detection assays included anti-BMAL1 [14], PER1, BMAL1-phospho-Ser90 (P-BMAL1-S90) and BMAL1-acetyl-Lys537 (Acetyl-BMAL1) previously generated in our laboratories [12,30], CK2β (Calbiochem, San Diego, CA, United States), CK2α, actin, RNA polymerase II (Santa Cruz. Biotechnology Inc., Santa Cruz, CA, US), Myc-tag (Upstate Biotechnology Inc., Lake Placid, NY, US), CLOCK (Affinity BioReagents, Golden, CO, US), CRY1 (kindly donated by Dr. Todo), CRY2 [14], His-tag, FLAF-tag (MBL Co. Ltd., Nagoya, Japan), and HRP-conjugated anti-rabbit/goat/mouse IgG (Zymed, South San Francisco, CA, US). Immunoblot data were quantified by computerized densitometry and statistical analysis were performed as described previously [12,14,38]. The density of the protein bands was normalized to actin levels and the value of the control sample was set as 1. Statistical analysis was performed using the Student’s *t* test. Calculated error bars indicate standard deviation (SD).

In vitro kinase assays were performed as described previously [12,13], using CK2 subunits, 1 mM ATP, and GST-BMAL1, with/without His-CRY1 (see below). CK2α, CK2β, and GST-BMAL1 were prepared as described previously [12]. Kinase activities were measured by immunoblot using an anti-P-BMAL1-S90 antibody, and quantified as described above. Immunoblot and kinase assay data were normalized to the control values.

GST-CK2α, α’, and β subunits were expressed in bacteria, purified and analyzed by CBB staining. Mammalian clock proteins labeled with ^35^S-methionine were produced using the TNT Quick Coupled Transcription/Translation system (Promega) with expression vectors for BMAL1 (donated by Dr. Ikeda) and V5-CRY1/2 (donated by Dr. Reppert). CK2 subunits and clock proteins were mixed (combinations as indicated in the text), incubated, and affinity-precipitated with glutathione Sepharose beads. Recombinant His (and V5)-tagged CRY1 protein was expressed in High Five insect cells using pIB/V5-His vector and InsectSelect System (Invitrogen, CA, US). His-CRY1 protein was purified using Talon-resin (Clontech, CA, US).

### Plasmid Construction

The plasmid vector pGL4-Per2, constructed to express the ~1.7 kb *mPer2* promoter-driven luciferase construct (*Per2-Luc*), has been described previously [12]. The vector pGL4-Bmal1 was constructed to express the ~1 kb *mBmal1* promoter-driven luciferase. The pcDNA (Invitrogen, Carlsbad, CA, US)-based vectors Myc-HA-mCRY1, Myc-mCRY2, FLAG-mCRY1 and the deletion mutants (*Cd1–4*; Fig 5C) were constructed to express *CMV* promoter-driven *CRY1/2*.

Retroviral expression vectors (pCLNCX) for the ~1.6 kb *mPer3* promoter [39]-driven luciferase (*Per3-Luc*) and the ~1 kb *mCry1* promoter-driven *Myc-CRY1* and *CMV*-promoter-driven *GFP* constructs have been described previously [12]. In addition, these experiments utilized previously prepared expression constructs for *mBmal1*-promoter-driven *BMAL1*-WT/mutants and *CMV*-promoter-driven *GFP* [4,12].

For the Split Luc complementation assay [23], the cDNA of Emerald Luciferase (ELuc) was obtained from Toyobo Co. Ltd. (Osaka, Japan). The multiple complement luciferase fragment cDNA construct (*McLuc1*) was generated as described previously [22]. The full-length mouse *BMAL1* and human *CK2β* cDNAs were ligated downstream of the C-terminal (*McLuc1* or *ELucC*) and N-terminal (*ELucN)* luciferase fragments, respectively (Fig 4A). Full-length *BMAL1*, *BMAL1* deletion mutants (*Bd1–5*; Fig 5A), and *CK2β* cDNAs, were ligated downstream of *ELucC and ELucN*, respectively. Each cDNA fragment was amplified by polymerase chain reaction (PCR) and inserted into pcDNA4/V5-His (B) or pcDNA3.1 (Invitrogen, Carlsbad, CA, US) vector backbones using multiple cloning sites. In the ELucC/McLuc1-BMAL1 expression vector, three repeats of the *BMAL1* Rev-erbA/ROR binding element (RRE) were added. To test promoter performance, the McLuc1-BMAL1 and ELucN-CK2β coding sequences were replaced by Luc (from pGL4), giving rise to the RREx3-CMV-Luc and CMV-Luc expression vectors. The sequences of all expression vectors were checked using a genetic analyzer (ABI310; Applied Biosystems, Foster City, CA, US). For long-lasting (stable) expression of the plasmids in human cells, episomal-type vectors were constructed by replacement of the CAG-promoter-MCS in pEBMulti-Hyg (Wako, Osaka, Japan) with CMV-ELucN-CK2β and RREx3-CMV-McLuc1-BMAL1 constructs. The expression vectors were purified with the PureLink HiPure Plasmid Filter Midiprep Kit (Invitrogen, Carlsbad, CA, US) prior to transfection of mammalian cells.

### Cell Culture, Real-Time Bioluminescence Analysis, and Data Processing

Established (NIH-3T3) and primary (MEF) mouse embryonic fibroblasts, and human osteosarcoma (U-2OS) cells were cultured as previously described [12,24,30,37]. BMAL1-deficient (KO) MEFs [7] were kindly donated by Dr. Bradfield. For clock synchronization, cells were treated with 0.1 μM dexamethasone (Dex) for 2 h. DNA transfection was performed using FuGENE HD according to manufacturer’s protocol (Roche Diagnostics Basel, Switzerland). The peptides BMs90p (RRDKMNSFIDELAS, a 14 amino acid BMAL1 peptide centered around BMAL1 Serine 90) and BMa90p (RRDKMNAFIDELAS, a negative control peptide with serine 90 replaced by alanine) were custom-made by Gen Script (Piscataway, NJ, US). Peptides were dissolved in water and applied to actively growing cultured cells at ≤60% confluence. The RetroMax expression system (Imgenex, San Diego, CA, US) was used to produce retrovirus for the rescue experiment. Retroviral infection was performed as previously described [12]. Real time bioluminescence activities were monitored using the Kronos Dio system (ATTO, Tokyo, Japan) as previously described [37].

All animal experiments were approved by the Toho University Animal Committee, and carried out under the control with Guidelines for Proper Conduct of Animal Experiments by Science Council of Japan. *mPER2* ^*Luc*^ mice (B6.129S6-*Per2* ^*tm1Jt*^/J)^21^ were purchased from Jackson Laboratories (Bar Harbor, ME, US) and maintained at 25°C on a 12 h light/dark (LD) cycle (light: zeitgeber time [ZT] 0–12; dark: ZT 12–24). Preparation of organotypic slices from 4–8 week old mice, real-time bioluminescence assay, and microscopic imaging analysis (using the LV200 Bioluminescence Imaging System; Olympus, Tokyo, Japan) and MetaMorph analysis (MetaMorph, Nashville, TN, US) were performed using previously published procedures [40], briefly described below. The reduction of the peak (Fig 1Bb) was quantified by evaluating the difference in peak values in Fig 1Ba over 2 d before and after BMs90p treatment. The reduction of rhythm amplitude (Fig 1Bb) was quantified by comparing peak and trough values in the detrended data (S3 Fig) over 2 d before and after BMs90p treatment.

Values obtained from bioluminescence analyses were normalized by the maximum peak intensities over time and further normalized by the averaged intensity over time, as described previously [37,41]. Real-time bioluminescence in cell cultures and organotypic slices treated with 0.2 mM Luciferin (Toyobo) were monitored using the Kronos Dio system with acquisition times of 2 (promoter-Luc assays) or 3 min (Split-Luc assays), according to the manufacturer’s protocol. Values were obtained from each sample in a given experiment using the same detectors. The *n*-values indicated for each experiment refer to the number of samples analyzed with the same detectors in the same experiments. The *y*-axis label “Bioluminescence” indicates that the relative photo-counting values reflect arbitrary units (a.u.) from raw data; “RLU” (Relative Light Units) indicates that the relative photo-counting values were normalized by averaging intensity over time. The *y*-axis label “Deviation from the moving average” indicates that the values were detrended according to the Kronos Dio instrument protocol (ATTO). In many cases, as indicated in the figure legends, detrended values were further normalized by averaging intensity over time. The data in these graph labeled "deviation from the moving average” were further normalized using maximum circadian peak intensities over time. Real-time bioluminescence for microscopic imaging was monitored using the LV200 microscope with acquisition times of 48 min (EM-gain = 400) according to the manufacturer’s protocol (Olympus). Values obtained from each tracked region of interest (ROI) surrounding neighboring small clusters of cell-areas were processed similarly.

### Statistical Analyses

We used factorial design analysis of *t* test to analyze data as appropriate. The data presented in this study represent the average of multiple experiments, as specified in the figure legends.

## Supporting Information

S1 Data Values for the quantitative experiments corresponding to main figures. Data from the quantitative/statistical analysis are shown with respect to the graphs in Figs 1–7. Data includes *p*-values for the statistical significance.
(XLSX)

S2 Data Values for the quantitative experiments corresponding to supporting figures. Data from the quantitative/statistical analysis are shown with respect to the graphs in S1–S5, S8 and S9 Figs. Data includes *p*-values for the statistical significance.
(XLSX)

S1 Fig Characterization of BMs90p. (A) BMAL1-IPs and lysates from untreated (-) and BMs90p (6 μM) treated NIH-3T3 cells were subjected to IB analysis for BMAL1, P-BMAL1-S90, CK2α, and CK2β, using actin as a loading control. Representative images of duplicate experiments are shown. (B) Clock performance and BMAL1 phosphorylation in NIH-3T3:Per2L cells after treatment with 6 μM BMs90p (S90p) and BMa90p (a peptide replaced Ser90 to Ala) for 30 min and subsequent clock-synchronization by dexamethasone (Dex) treatment for 30 min. (a) Cell cultures were monitored for luciferase activity by real-time bioluminescence assay. Representative raw (left) and detrended/averaged (right) profiles are shown (*n* = 3). (b) Immunoblot (IB) analysis of BMAL1-IP and lysates from untreated (-) and S90p/A90p (6 μM) treated cells for BMAL1, P-BMAL1-S90 (Ser90-phoshorylated BMAL1), CK2α, CK2β, and actin (loading control).
(EPS)

S2 Fig Effect of BMs90p-treatment on circadian rhythms in PER2::LUC liver and SCN organotypic slices. Clock performance of organotypic slices of liver (A–C) and SCN (D–F) from *mPER2* ^*Luc*^ mice following treatment with 6 μM BMs90p or mock treatment with fresh medium was monitored by real-time bioluminescence imaging. Treatment was performed around the PER2::LUC trough (A, B: liver; ~CT5, D, E: SCN; ~CT1) or peak (C: liver; ~CT17, F: SCN; ~CT13) phase. Note that reduction of peak bioluminescence after BMs90p-treatment was only observed when slices were treated around the trough phase.
(EPS)

S3 Fig Reduction of amplitudes in circadian rhythms in liver and SCN after BMs90p-treatment. As S2 Fig, except that data were further normalized using maximum circadian peak intensities over time. A reduction of rhythm amplitude (Fig 1Bb) after BMs90p-treatment was calculated from detrended data by comparing averaged differences between the peak and trough over 2 d before and after the treatment. Similarly, reduction of peak bioluminescence (Fig 1Cb) after BMs90p-treatment was calculated from raw data (Fig 1Ca) by comparing averaged peak differences over 2 d pre- and post-treatment.
(TIF)

S4 Fig Reduction of Per2L-bioluminescence in SCN neurons after BMs90p-treatment. An organotypic SCN slice from *mPER2* ^*Luc*^ mice was monitored by real-time bioluminescence microscopy (LV200, Olympus, Japan). Cultures were treated with 6 μM BMs90p around trough phase (CT2) as indicated. (A) Bioluminescence recordings of 24 different cell regions. (B) Representative images of Per2L SCN slices around the trough (CT2) and peak phase (CT14), recorded for 6 d. Fig 1Ca shows normalized, detrended plots.
(EPS)

S5 Fig Restoration of circadian gene expression in CRY1/2-deficient MEFs by rhythmic CRY1 expression. Cry1/2^-/-^ MEFs, expressing the Per3-Luc clock reporter gene and either CMV-promoter-driven GFP (A) or mCry1 promoter-driven Myc-CRY1 (B), were Dex-synchronized and monitored by real-time bioluminescence recording. For each graph, curves represent detrended values, normalized against the maximum value (set as 1).
(EPS)

S6 Fig CK2 subunits binds to BMAL1 and CRY1/2 proteins. (A) Differential binding of CK2 subunits to the mammalian clock proteins in vitro. Purified bacterially expressed GST-CK2α, α′, and β subunits (upper panel) were 1:1 mixed with in vitro translated ^35^S-methionine labeled BMAL1, V5-CRY1, or V5-CRY2, affinity purified using glutathione Sepharose beads. The precipitates were subjected to SDS-PAGE and visualized by autoradiography. (B) CK2β forms complex with CRY1/2. Shown is a representative example of a coimmunoprecipitation experiment with endogenous CK2β and CRY1 or CRY2 in BMAL1-deficient MEFs. Lysates and CK2β- and CRY1/2- IPs were subjected to IB analysis for CK2β and CRY1/2.
(EPS)

S7 Fig Circadian BMAL1–CK2β association shows inversed-phase to circadian CK2α-mediated BMAL1-S90 phosphorylation. WT, *Cry1/2* ^−/−^ MEFs, and *Cry1/2* ^−/−^ MEFs stably expressing *mCry1* promoter-driven Myc-CRY1 or CMV-promoter-driven Myc-GFP (control) were Dex-synchronized. BMAL1 and CK2β IPs and total lysates were subjected to IB analysis for BMAL1, P-BMAL1-S90, CK2α/β, and CRY1/2, using PER1/actin as a loading control.
(EPS)

S8 Fig Circadian BMAL1–CK2β binding rhythms in living cells. (A) Circadian interaction between BMAL1–CK2β in Dex-synchronized ELucN-CK2β and McLuc1-BMAL1 expressing U-2OS cells was monitored by the real-time Split Luc assay. Circadian profiles obtained in three independent experiments (green, light blue, and dark blue) are shown. Data were normalized by setting the value of the first peak as 1. (B) Prolonged circadian monitoring of the interaction between BMAL1 and CK2β in U-2OS cells transfected with pEBMulti-based episomal type expression vectors suitable for longer term detection (Wako, Japan) for ELucN-CK2β and McLuc1-BMAL1. Representative detrended/averaged circadian profiles (averaged Period = 25.8 h, Acrophase = 15.7 h, as determined using Cosinor and Acro software; http://www.circadian.org/softwar.html) are shown with the maximum normalized values set as 1. (C) Analysis of RREx3/CMV and CMV promoters, as used for the ectopic expression of BMAL1 and CK2β in the Split Luc assay. Dex-synchronized U-2OS cells, expressing RREx3/CMV (for BMAL1 in the split Luc vectors) or CMV (for CK2β in the split Luc vectors)-promoter driven luciferase were monitored by real-time bioluminescence imaging. Circadian profiles (averaged values from *n* = 3 experiments) were normalized, with the maximum value set as 1. Note that RREx3/CMV-Luc activity did not show evident circadian oscillation.
(EPS)

S9 Fig Ectopic expression of BMAL1 and CK2β from split Luc vectors does not affect endogenous circadian phase. Dex-synchronized U-2OS cells, stably expressing *Bmal1*-promoter driven luciferase in the absence (black line) or presence (blue line) of ELucN-CK2β and McLuc1-BMAL1 (Split Luc assay for BMAL1–CK2β association; same dose as used in Fig 4C) were monitored by real-time bioluminescence imaging. Circadian profiles (averaged values from *n* = 5 experiments) were normalized, with the maximum value set as 1. Note that Split Luc activity can be neglected because of the much higher *Bmal1*-luciferase activity.
(EPS)

S10 Fig Acetylation-mediated BMAL1-Ser90 phosphorylation is not prerequisite for BMAL1-Ser90 phosphorylation. Mutation of BMAL-Lys537, the CLOCK-mediated acetylation site, does not erase BMAL1-Ser90 phosphorylation. *Bmal1* ^−/−^ MEFs stably expressing *mBmal1* promoter-driven Myc-BMAL1-WT (wild type), Myc-BMAL1-K537R (CLOCK-mediated acetylation site-deficient mutant) and CMV-promoter-driven Myc-GFP (control) were Dex-synchronized. The supernatants from the cells at 22 h after treatment were subjected to Myc-BMAL1 IP. IPs and lysates were subjected to IB analysis for Myc-BMAL1/GFP, P-BMAL1-S90, Acetyl-BMAL1, CK2β, and CRY1/2.
(EPS)

S11 Fig Nuclear dominant localization of P-BMAL1-S90. WT MEFs were synchronized with dexamethasone and subsequently harvested at 4 h intervals. For each time point, BMAL1-IPs and lysates from the nuclear and cytoplasmic fractions (prepared as previously described [38]) were subjected to IB analysis for P-BMAL1-S90 and BMAL1, using RNA polymerase II (Pol II) and actin as the loading controls. Red and black arrows indicate the position of major P-BMAL1-S90 (~90 kDa) and unmodified BMAL1 (~70 kDa), respectively.
(EPS)

S12 Fig Effect of CRY1 protein on the in vitro CK2α-mediated BMAL1-S90 kinase activity. (A) Purified recombinant His-CRY1 protein expressed in High Five insect cells can bind to BMAL1. His-CRY1 expressed in High Five cells was purified and detected by CBB-staining and immunoblotting with anti-His antibody. By IB analysis for the His- immunoprecipitate (His-IP) for the mixture of the purified GST-BMAL1 and His-CRY1, GST-BMAL1 and His-CRY1 were detected with anti-BMAL1 and anti-His antibodies. Note that no apparent band with higher molecular weight than His-CRY1 was detected in the CBB-stained gel. (B) His-CRY1 does not show inhibitory effect on the in vitro BMAL1-S90 phosphorylation. CK2α-mediated BMAL1-S90 kinase activity was measured by in vitro kinase assay with GST-BMAL1, CK2α, and differential doses of His-CRY1 and CK2β proteins, under the procedure for Fig 2C.
(EPS)

S1 Movie Effect of BMs90p-treatment on Per2L-rhythm in the SCN organotypic slice. An organotypic slice of the SCN from *mPER2* ^*Luc*^ mice was monitored by real-time bioluminescence microscopy (LV200, Olympus, Japan). Cultures were treated with 6 μM BMs90p around the trough phase (at time Zero after 2 d and 17 h) as indicated in Fig 1C and S4 Fig.
(MP4)

## Abbreviations

BMAL1-P: BMAL1-Ser90 phosphorylation
CCGs: clock-controlled output genes
CK2: Casein Kinase-2
Cry: Cryptochrome
HDACs: histone deacetylases
IB: immunoblot
IP: immunoprecipitates
MEFs: mouse embryonic fibroblasts
NR1D1 (also known as Rev-Erbα): nuclear receptor subfamily 1 group D member 1
Luc: luciferase
PAC: PAS-associated C-terminal
Per: period
Ror: Retinoic acid receptor related orphan receptor
SCN: SupraChiasmatic Nuclei
WT: wild type
