# Supplementary figures and images for: CRY Drives Cyclic CK2-Mediated BMAL1 Phosphorylation to Control the Mammalian Circadian Clock
Source: PLoS Biol. 2015 Nov 12;13(11):e1002293. doi: 10.1371/journal.pbio.1002293 (PMC4642984; doi:10.1371/journal.pbio.1002293)

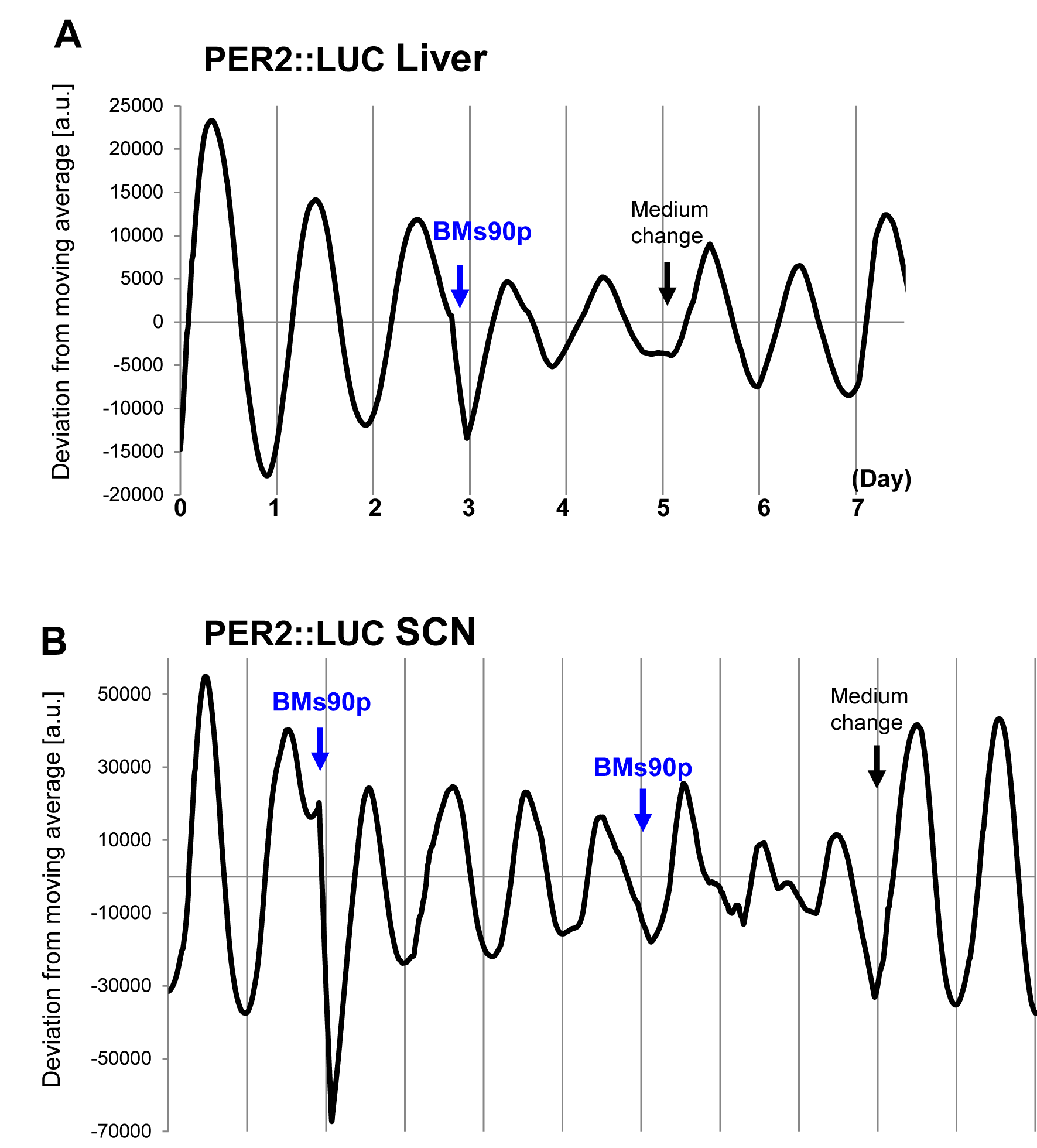

Supplement: S3 Fig — As S2 Fig, except that data were further normalized using maximum circadian peak intensities over time. A reduction of rhythm amplitude (Fig 1Bb) after BMs90p-treatment was calculated from detrended data by comparing averaged differences between the peak and trough over 2 d before and after the treatment. Similarly, reduction of peak bioluminescence (Fig 1Cb) after BMs90p-treatment was calculated from raw data (Fig 1Ca) by comparing averaged peak differences over 2 d pre- and post-treatment. (TIF) [file pbio.1002293.s005.tif]
